# Introduction of an adhesion factor to *cube in cube* models and its effect on calculated moduli of particulate composites

**DOI:** 10.1038/s41598-022-20629-2

**Published:** 2022-09-28

**Authors:** Julian Rech, Esther Ramakers–van Dorp, Patrick Michels, Bernhard Möginger, Berenika Hausnerova

**Affiliations:** 1 Bonn-Rhein-Sieg University of Applied Sciences, Von-Liebig-Straße 20, 53359 Rheinbach, Germany; 2 Faculty of Technology, Tomas Bata University in Zlin, Vavreckova 275, 76001 Zlin, Czech Republic; 3 Centre of Polymer Systems, University Institute, Tomas Bata University in Zlin, nam. T.G. Masaryka 5555, 76001 Zlin, Czech Republic

**Keywords:** Engineering, Materials science

## Abstract

The cube in cube approach was used by Paul and Ishai-Cohen to model and derive formulas for filler content dependent Young’s moduli of particle filled composites assuming perfect filler matrix adhesion. Their formulas were chosen because of their simplicity, and recalculated using an elementary volume approach which transforms spherical inclusions to cubic inclusions. The EV approach led to expression of the composites moduli that allows introducing an adhesion factor *k*_adh_ ranging from 0 and 1 to take into account reduced filler matrix adhesion. This adhesion factor scales the edge length of the cubic inclusions, thus reducing the stress transfer area between matrix and filler. Fitting the experimental data with the modified Paul model provides reasonable *k*_adh_ for PA66, PBT, PP, PE-LD and BR which are in line with their surface energies. Further analysis showed that stiffening only occurs if *k*_adh_ exceeds $\sqrt{{E}_{\mathrm{M}}/{E}_{\mathrm{F}}}$ and depends on the ratio of matrix modulus and filler modulus. The modified model allows for a quick calculation of any particle filled composites for known matrix modulus *E*_M_, filler modulus *E*_F_, filler volume content *v*_F_ and adhesion factor *k*_adh_. Thus, finite element analysis (FEA) simulations of any particle filled polymer parts as well as materials selection are significantly eased. FEA of cubic and hexagonal EV arrangements show that stress distributions within the EV exhibit more shear stresses if one deviates from the cubic arrangement. At high filler contents the assumption that the property of the EV is representative for the whole composite, holds only for filler volume contents up to 15 or 20% (corresponding to 30 to 40 weight %). Thus, for vast majority of commercially available particulate composites, the modified model can be applied. Furthermore, this indicates that the *cube in cube* approach reaches two limits: (i) the occurrence of increasing shear stresses at filler contents above 20% due to deviations of EV arrangements or spatial filler distribution from cubic arrangements (singular), and (ii) increasing interaction between particles with the formation of particle network within the matrix violating the EV assumption of their homogeneous dispersion.

## Introduction

Sustainability demands on performance of polymer parts are steadily increasing. Not only polymeric systems from renewable sources are currently developed, but contemporary established materials commodities are recycled. To meet these requirements, polymers are often modified by blending, copolymerization and reinforcement with particulate fillers and fibers. The access of these materials to emerging applications brings necessity of fast availability of relevant physical properties as stiffness, strength, and thermal properties e.g. thermal length expansion or heat conductivity.

In this perspective, use of reinforced composites will increase substantially^1^, and their performance has to be determined with respect to varying or even undefined mechanical properties of raw materials. This can be achieved by an elaborate thermo-mechanical analysis of a particular composite system (which is expensive and time consuming) or via accurate predictions of composite properties using analytical approaches.

Performance of composites is determined by properties of a matrix, dispersed phase and interface between filler and matrix^2^. A filler-matrix adhesion can be modified by coupling agents. He and Jiang^3^ showed that glass bead reinforced polyepichlorhydrin pretreated with a coupling agent yielded in an enhanced stiffness behavior over the whole filler volume range (5–35%). Demir et al.^4^ investigated the effect of different coupling agents on the performance of luffa fiber/polypropylene composites and found out that the moduli of the composites containing 15 wt% fiber increased by 52 to 98% after modification. They concluded that the addition of the coupling agents was accompanied by the decrease in water absorption due to a better adhesion between fibers and matrix. Similar conclusion was presented by Jacob et al.^5^, which studied the effect of various silane coupling agents on viscoelastic properties of sisal/oil palm hybrid fiber reinforced natural rubber composites, and reported that the chemical modification of fibers with respect to the used coupling agents led to improved wettability and consequently to an increase in storage and loss moduli of the composites.

Ku et al.^6^ reviewed an effect of various pretreatments and coupling agents on tensile properties of natural fiber reinforced polymer composites. They summarized the studies, which confirmed the increase in Young’s modulus due to the enhanced interfacial adhesion caused by the pretreatment of the fillers and the coupling agents. On the other hand, Dekkers et al.^7^ and Dibendetto et al.^8^ used glass beads as fillers for polystyrene and epoxy resin matrices and found that the Young’s moduli were not affected by the surface treatment. Wang et al.^9^ observed increasing moduli of polypropylene/barium sulfate composites using stearic acid, silane and maleic anhydride as surface modifications, but they attributed it not to the improved interfacial adhesion, but rather to higher crystallinity and the formation of the crystal lattices in the matrix.

Numerous models have been developed to calculate Young’s moduli of particulate polymer composites considering elastic properties of fillers and matrices, volume content and aspect ratio, Table 1. For modelling purposes, a two-phase model in terms of the representative volume element is subjected to unidirectional stresses and strains for calculating expressions of elastic constants^10,11^. Three-phase models are not considered in this work because their higher complexity requires additional materials parameters^12–14^.

**Table 1:** Summary of two-phase models to predict Young’s moduli of particulate composites.

Models	Young’s modulus of particle filled composite
Voigt^15^	${E}_{C}={E}_{M}\left(\left(1-{v}_{F}\right)+ \frac{{E}_{F}}{{E}_{M}}{v}_{F}\right)={E}_{M}\left(1-{v}_{F}\right)+ {E}_{F}{v}_{F}$ (1)
Reuss^16^	${E}_{C}={E}_{M}\frac{\frac{{E}_{F}}{{E}_{M}}}{{v}_{F} + \frac{{E}_{F}}{{E}_{M}}\left(1-{v}_{F}\right)}= \frac{{E}_{F}{E}_{M}}{{E}_{F}\left(1-{v}_{F}\right)+{E}_{M}{v}_{F}}$ (2)
Guth^17^	${E}_{C}={E}_{M}\left(1+{K}_{E}{v}_{F}+14.1{v}_{F}^{2}\right)$(3)
Paul^20^	${E}_{C} ={E}_{M}\left(\frac{1+\left(\frac{{E}_{F}}{{E}_{M}}-1\right){v}_{F}^\frac{2}{3}}{1+\left(\frac{{E}_{F}}{{E}_{M}}-1\right)\left({v}_{F}^\frac{2}{3}-{v}_{F}\right)}\right)$(4)
Hirsch^22^	${E}_{C}={E}_{M} \left(\chi \left(\left(1-{v}_{F}\right)+ \frac{{E}_{F}}{{E}_{M}}{v}_{F}\right)+ \right) +\frac{\left(1-\chi \right) \frac{{E}_{F}}{{E}_{M}}}{{v}_{F} + \frac{{E}_{F}}{{E}_{M}}\left(1-{v}_{F}\right)}$(5)
Counto^23^	${E}_{C} ={E}_{M}\left( \frac{\left(1-{v}_{F}^\frac{1}{2}\right)+{v}_{F}^\frac{1}{2}\frac{{E}_{F}}{{E}_{M}}}{{\left(1-{v}_{F}^\frac{1}{2}\right)}^{2}+\left(1-{v}_{F}^\frac{1}{2}\right) {v}_{F}^\frac{1}{2}\frac{{E}_{F}}{{E}_{M}}+{v}_{F}^\frac{1}{2}}\right)$(6)
Takayanagi et al.^19^	${E}_{C} ={E}_{M}/\left(\frac{\frac{5{v}_{F}}{\left(2+3{v}_{F}\right)}}{\left(1-\frac{\left(2+3{v}_{F}\right)}{5}\right)+\frac{\left(2+3{v}_{F}\right)}{5}\frac{{E}_{F}}{{E}_{M}}}+\frac{1-\frac{5{v}_{F}}{\left(2+3{v}_{F}\right)}}{\frac{{E}_{F}}{{E}_{M}}}\right)$(7)
Ishai-Cohen^21^	${E}_{C} ={E}_{M}\left(\frac{ \frac{{E}_{F}}{{E}_{M}}+ \left(\frac{{E}_{F}}{{E}_{M}}-1\right) \left({v}_{F}- {v}_{F}^\frac{1}{3} \right)}{\frac{{E}_{F}}{{E}_{M}}- \left(\frac{{E}_{F}}{{E}_{M}}-1\right){v}_{F}^\frac{1}{3}}\right)$(8)
Halpin–Tsai^24^	${E}_{C} ={E}_{M}\left(\frac{1+\frac{\left(\frac{{E}_{F}}{{E}_{M}}-1\right)}{\left(\frac{{E}_{F}}{{E}_{M}}+\xi \right)}\xi {v}_{F}}{1-\frac{\left(\frac{{E}_{F}}{{E}_{M}}-1\right)}{\left(\frac{{E}_{F}}{{E}_{M}}+\xi \right)}{v}_{F}}\right)$(9)
Nielsen^25,27^	${E}_{C} ={E}_{M}\left(\frac{1+({K}_{E}-1)\left(\frac{\frac{{E}_{F}}{{E}_{M}}-1}{\frac{{E}_{F}}{{E}_{M}}+({K}_{E}-1) }\right){ v}_{F}}{1-\left(\frac{\frac{{E}_{F}}{{E}_{M}}-1}{\frac{{E}_{F}}{{E}_{M}}+\left({K}_{E}-1\right) }\right)\left(1+\frac{\left(1-{v}_{F,max}\right){ v}_{F}}{{v}_{F,max}^{2}}\right) {v}_{F}}\right)$(10)

Moduli of matrix E_*M*_ and filler E_*F*_, filler volume content v_*F*_, parameter χ determining stress transfer between fiber and matrix, geometry factor ξ, Einstein coefficient KE and maximum volume fraction v_F,max_.

The models of Voigt^15^ and Reuss^16^ represent the upper bound for the uniform stress distribution and the lower bound for the uniform strain distribution, respectively, and differ significantly. In most cases the measured Young’s moduli lie in between. This indicates that they can only serve as a rough estimation, and that real loading states are more complex than uniform stress or uniform strain. Guth^17^ proposed a two-phase model based on Einstein’s approach^18^ to determine the viscosity of a suspension with spherical inclusions.

The *cube in cube* models (4–10) take into account a dispersed structure of particle filled composites. Takayanagi et al.^19^ combined the models of Voigt and Reuss in a way that allows for introducing a dependency on filler volume content. Paul^20^ assumed uniform stress states with a perfect filler-matrix adhesion, whereas Ishai and Cohen^21^ assumed uniform strain states. Their models represent more precise upper and lower bounds for composites moduli than those of Voigt and Reuss. More complicated models require additional parameters, which are determined by a fitting procedure:

- Hirsch^22^ combined the models of Voigt and Reuss balancing their contributions with a factor *χ.*
- Counto^23^ developed a simple model for concrete systems assuming perfect filler matrix adhesion; it coincides with the Hirsch model for *χ* = 0.5.
- Halpin and Tsai^24^ developed a model to describe anisotropic properties of fiber and filler reinforced composites with one equation in which an efficiency factor *ξ* takes into account fiber or filler geometry and spatial orientation of fibers or platelets; due to its simplicity it became popular regardless of limited accuracy.
- Nielsen^25^, Lewis and Nielsen^26^ and Nielsen^27^ proposed an alternative model based on Halpin–Tsai^24^ and Kerner^28^ in which mechanical properties depend additionally on fiber or filler geometry and load direction, maximum filler content *v*_F,max_; moreover, Nielsen assumed a Poisson ratio-dependent Einstein coefficient *K*_E_.

Moduli of matrix E_*M*_ and filler E_*F*_*,* filler volume content v_*F*_*,* parameter *χ* determining stress transfer between fiber and matrix, geometry factor *ξ,* Einstein coefficient *K*_E_ and maximum volume fraction *v*_F,max_.

All models depicted in Table 1 assume a perfect filler-matrix adhesion. Reduced adhesion between a filler and a matrix has not been considered yet in *cube in cube* models. The aim of this study is to introduce an adhesion factor having values between “0” and “1”to the EV models of Paul and Ishai-Cohen to express adhesion quantitatively, and to investigate how the adhesion factor and spatial arrangement of EV affect the filler content dependent composites moduli.

## Theoretical considerations

**Figure 1:**
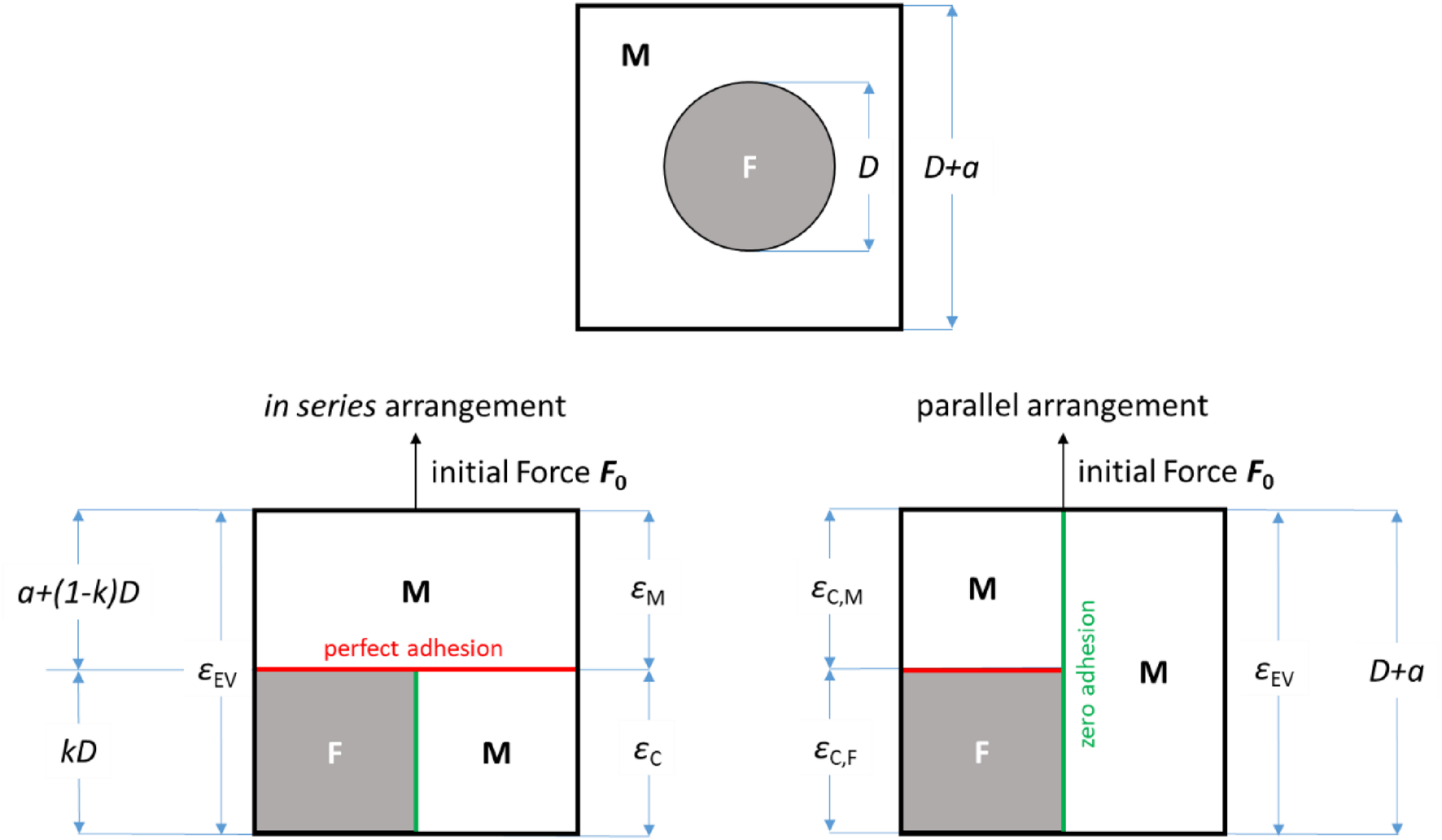
Cubic elementary volume (EV) containing a spherical inclusion with diameter *D* and distance *a* between inclusions (top), and its *cube in cube* consideration *in series* and *parallel* arrangements with adhesion boundary conditions.

If filler particles are homogeneously dispersed in a matrix, one can define an elementary volume (EV) containing a single particle that is to be representative for composite properties. A cube of length *D* + *a* is shown with a spherical inclusion of diameter *D* consisting of matrix M and filler, Fig. 1 top. The “cube in cube” approach requires that a particle of any geometry is transformed to a cube of length *k D* with the efficiency factor *k*. It takes into account that less than the maximum cross-section contributes to the stress transfer, and thus depends on particle shapes. For spheres it is determined by the condition

11 $${V}_{sphere}= \frac{\pi }{6} {D}^{3}= {k}^{3} {D}^{3}= {V}_{cube} \Rightarrow k= {k}_{sphere} = \sqrt[3]{\frac{\pi }{6}} \cong 0.81$$

with volume of sphere ${V}_{sphere}$ and volume of cube ${V}_{cube}$. In order to calculate the filler volume content dependent Young’s modulus of the composite, the cube has to be divided into a matrix part and a composite part done either *in series* or *parallel* to account for microscopic mechanical properties, Fig. 1 bottom left and right.

This allows calculation how the modulus of the EV depends on the arrangements of matrix part and composites part. Furthermore, the analysis of the EV separation shows that certain assumptions were made with respect to the adhesions acting between matrix and filler:

- For the *in series* arrangement “perfect” adhesion is assumed over the entire cross-section (*D* + *a*)^2^ perpendicular to the load direction between matrix part and composites part, and “zero” adhesion is required between filler and matrix of the composite part along the load direction. If the EV cube is strained to *ε*_EV_ by the load *F*_0_, the matrix part experiences the strain *ε*_M_ and the composites part the strain *ε*_C_.
- For the *parallel* arrangement “perfect” adhesion is assumed only over the cross-section (*k D*)^2^ of the composite part between the filler and the matrix perpendicular to the load direction, and “zero” adhesion is required between the composite part and the matrix part along the load direction. If the EV cube is strained to *ε*_EV_ by the load *F*_0_, the matrix of the composite part experiences the strain *ε*_C,M_ and the filler of the composite part the strain *ε*_C,F_.

If the aspect ratio is not close to “1”, one has to distinguish the extremes “along the long axis” and “perpendicular to the long axis” e.g. with a square column consideration. In this consideration a reduction of adhesion can only apply to “perfect” adhesion, whereas “zero” adhesion leads to neglecting of shear stresses.

### Case 1: in series EV arrangement and Paul model

The first step is to define the elastic stress–strain-relation of the EV to represent the macroscopic mechanical behavior:

12 $$\sigma = {\sigma }_{EV}= {E}_{EV }\frac{\Delta (D+a)}{D+a}= {E}_{EV} {\varepsilon }_{EV }\Rightarrow {\varepsilon }_{EV}= \frac{\sigma }{{E}_{EV}}$$

with external stress $\sigma$, stress acting on EV ${\sigma }_{EV}$, distance $a$ between particles, modulus of EV ${E}_{EV}$ and strain of EV ${\varepsilon }_{EV}$. The second step is to define the stress–strain-relation of the matrix part with respect to the microscopic strain ${\varepsilon }_{M}$ of the matrix part:

13 $$\sigma = {\sigma }_{M}= {E}_{M }\frac{\Delta a}{a}= {E}_{M} {\varepsilon }_{M }\Rightarrow {\varepsilon }_{M}= \frac{\sigma }{{E}_{M}}$$

with stress acting on matrix part ${\sigma }_{M}$, matrix modulus ${E}_{M}$, and matrix strain ${\varepsilon }_{M}$, and the stress–strain-relation of the composites part:

14 $$\sigma = {\sigma }_{C}= {\sigma }_{C, M}+ {\sigma }_{C, F} \Rightarrow {\varepsilon }_{C}= \frac{\sigma }{{E}_{C}}$$

with stress acting on composite ${\sigma }_{C}$, stress acting on matrix of composites part ${\sigma }_{C, M}$, stress acting on filler of composites part ${\sigma }_{C, F}$, strain of composites part ${\varepsilon }_{C}$, and the composites modulus ${E}_{C}$. The stress acting on the matrix of the composites part is given by:

15 $${\sigma }_{C, M}= \left(1- \frac{{(k D)}^{2}}{{(D+a)}^{2}}\right) {E}_{M} {\varepsilon }_{C},$$

and the stress acting on the filler of the composites part is given by:

16 $${\sigma }_{C, F}= \frac{{(k D)}^{2}}{{(D+a)}^{2}} {E}_{F} {\varepsilon }_{C}$$

with filler modulus ${E}_{F}$. Introduction of (15) and (16) in (14) links the external stress to the microscopic strain ${\varepsilon }_{C}$ yielding

17 $$\sigma  = \sigma _{C}  = \left( {\left( {\underbrace {{1 - \frac{{\left( {KD} \right)^{2} }}{{(D + a)^{2} }}}}_{\begin{subarray}{l}    {\text{relative}}\;{\text{matrix}} \\    {\text{cross-section}}  \end{subarray} }} \right)E_{M}  + \underbrace {{\frac{{\left( {KD} \right)^{2} }}{{(D + a)^{2} }}}}_{\begin{subarray}{l}    {\text{relative}}\;{\text{filler}} \\    {\text{cross-section}}  \end{subarray} }} \right)\varepsilon _{C} .$$

If the interfacial adhesion is not perfect, the stress transfer to the inclusion is reduced. The structure of (17) allows to identify where non-perfect reduced filler matrix adhesion may come into play as it has to decrease the stiffening capability of the filler. Thus, the second term in the brackets on the right side of (17) describing the contribution of filler modulus *E*_F_ to the stress–strain-relation of the composites does not contribute to full extent. In this context the EV representation of stress–strain-relation (17) provides a hint, where to introduce the dimensionless adhesion factor *k*_adh_ that ranges from “0” (no adhesion) to “1” (perfect adhesion). In Eq. (17) one deals with the coefficients attached to *E*_M_ and *E*_F_ which represent relative cross-sections of matrix and filler, respectively. Therefore, the adhesion factor is introduced quadratically as it is to reduce the edge length of the filler cube. In that respect the physical meaning of the adhesion factor is that it scales the edge length of the filler cube, and thus the available contact area of stress transfer between filler and matrix to account for the reduced adhesion due to the surface energies of filler and matrix, respectively. After elimination of the particle diameter *D*, one gets:

18 $$\sigma \underbrace { = }_{{d = \frac{a}{D}}} \left(\left(1- \frac{{k}^{2}}{{\left(1+d\right)}^{2}}\right) {E}_{M} + {k}_{adh}^{2} \frac{{k}^{2}}{{\left(1+d\right)}^{2}} {E}_{F}\right){\varepsilon }_{C}$$

Solving (18) for *ε*_C_ yields the strain of the composite part.

19 $${\varepsilon }_{C}=\frac{\sigma }{{E}_{M} \left(1 + \frac{{k}^{2}}{{\left(1+d\right)}^{2}} \frac{{k}_{adh}^{2}\boldsymbol{ }{E}_{F}-{E}_{M} }{{E}_{M}}\right)}$$

The strain of the EV *ε*_EV_ depends on the strains of both the matrix part *ε*_M_ and the composites part *ε*_C_ (see Appendix A):

20 $${\varepsilon }_{EV}\left({\varepsilon }_{M}, {\varepsilon }_{C}\right)= \left(1- \frac{k}{\left(1+d\right)}\right) {\varepsilon }_{M}+ \frac{k}{(1+d)} {\varepsilon }_{C}$$

Substituting the strains from (12), (13) and (19) in (20), and solving for *E*_EV_ yields:

21 $${E}_{EV}= {E}_{M} \left(1+ \frac{\frac{{(k)}^{3}}{{(1+d)}^{3}} \frac{{k}_{adh}^{2}\boldsymbol{ }{E}_{F}-{E}_{M} }{{E}_{M}}}{1 + \frac{{(k)}^{2}}{{(1+d)}^{2}} \frac{{k}_{adh}^{2}\boldsymbol{ }{E}_{F}-{E}_{M} }{{E}_{M}} - \frac{{(k)}^{3}}{{(1+d)}^{3}} \frac{{k}_{adh}^{2}\boldsymbol{ }{E}_{F}-{E}_{M} }{{E}_{M}}}\right)$$

The efficiency factor *k* is related to the filler volume content *v*_F_ by

22 $${v}_{F}= \frac{{(k )}^{3}}{{(1+d)}^{3}}; {{v}_{F}}^{2/3}= \frac{{(k)}^{2}}{{(1+d)}^{2}}; {v}_{F}^{1/3}= \frac{k}{\left(1+d\right)},$$

and (21) becomes:

23 $${E}_{EV}= {E}_{M}\left(1 + \frac{{v}_{F} \frac{{k}_{adh}^{2}\boldsymbol{ }{E}_{F}-{E}_{M} }{{E}_{M}}}{1 + {{v}_{F}}^{2/3} \frac{{k}_{adh}^{2}\boldsymbol{ }{E}_{F}-{E}_{M} }{ {E}_{M}} - {v}_{F} \frac{{k}_{adh}^{2}\boldsymbol{ }{E}_{F}-{E}_{M} }{ {E}_{M}}} \right)$$

For perfect adhesion with *k*_adh_ = 1, Eq. (23) becomes identical to Paul’s relation in Table 1.

**Figure 2:**
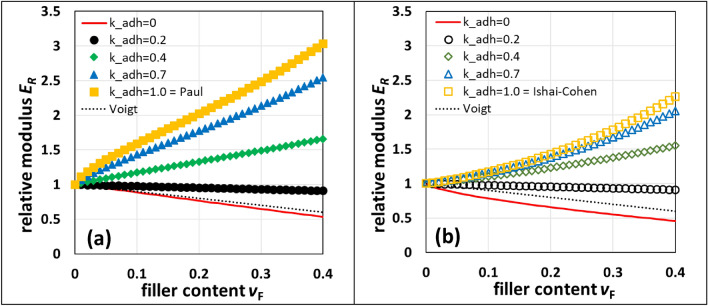
Calculated filler volume content dependent relative moduli *E*_R_ for different adhesion factors *k*_adh_ with *E*_M_ = 3200 MPa and *E*_F_ = 63,000 MPa for (**a**) *in series* EV arrangement and (**b**) *parallel* EV arrangement. The dotted line represents the decreasing *E*_R_ for a foam with *E*_F_ = 0 MPa according to Voigt.

From Eq. (23) follows that a stiffening effect only occurs if ${k}_{adh}> \sqrt{{E}_{M}/{E}_{F}}$. Calculating the filler volume content dependent relative modulus *E*_R_ = E_C_/E_M_ with *E*_M_ = 3200 MPa and *E*_F_ = 63,000 MPa, respectively, and varying *k*_adh_ between 0 and 1 shows that for *k*_adh_ > 0.23 one gets stiffening, and for *k*_adh_ < 0.23 softening, Fig. 2a.

### Case 2: parallel EV arrangement and Ishai-Cohen model

The external stress σ acts macroscopically on the EV according to (12). The stress–strain-relation of the matrix part is given by

24 $$\sigma = {\sigma }_{M} = {E}_{ M} {\varepsilon }_{EV}\Rightarrow {\varepsilon }_{EV}= \frac{{\sigma }_{M}}{{E}_{M}},$$

and the stress–strain-relations of the composites part are given by

25a $$\sigma ={\sigma }_{C}= {{E}_{ C} \varepsilon }_{C}= {{E}_{ C} \varepsilon }_{EV}$$

25b $$\mathrm{with } \; \; \sigma ={\sigma }_{C,M}= {E}_{ M} {\varepsilon }_{C,M}$$

25c $${\sigma =\sigma }_{C, F}={{k}_{adh}^{2} E}_{ F} {\varepsilon }_{C,F}$$

In (25c) the adhesion factor k_ash_ is already introduced to account for possibly reduced load transfer from matrix to filler. The external stress *σ* is distributed on the matrix part by ${\sigma }_{M}$ and the composites part by ${\sigma }_{C}$ due to the corresponding cross-sections yielding

26 $$\sigma = {\sigma }_{M}\frac{{A}_{M}}{{A}_{EV}}+ {\sigma }_{C}\frac{{A}_{C}}{{A}_{EV}} = {\sigma }_{M}\left(1-\frac{{k}^{2}}{{\left(1+d\right)}^{2}}\right)+ {\sigma }_{C}\frac{{k}^{2}}{{\left(1+d\right)}^{2}}$$

With cross-section of matrix A_M_, cross-section of composites part A_C_, and cross-section A_EV_ of EV. The strains of ${\varepsilon }_{EV}$, ${\varepsilon }_{C,M}$ and ${\varepsilon }_{C,F}$ of the composites part are connected by

27 $${\varepsilon }_{EV}= \left(1-\frac{k}{(1+d)}\right) {\varepsilon }_{C,M} + \frac{k}{(1+d)} {\varepsilon }_{C,F}$$

Insertion of (25a), (25b) and (25c) in (27) and solving for *E*_C_ yields

28 $${E}_{EV}={E}_{ M} \left(1+\frac{\frac{{k}^{3}}{{\left(1+d\right)}^{3}} \left({k}_{adh}^{2}\boldsymbol{ }{E}_{F} - {E}_{M}\right)}{ {k}_{adh}^{2}\boldsymbol{ }{E}_{F} - \frac{k}{1+d} \left({k}_{adh}^{2}\boldsymbol{ }{E}_{F} - {E}_{M}\right)}\right)$$

or in terms of filler volume content *v*_*F*_ using (22)

29 $${E}_{EV}={E}_{ M} \left(1+\frac{{v}_{F} \frac{{k}_{adh}^{2}\boldsymbol{ }{E}_{F} - {E}_{M}}{{E}_{M}}}{ \frac{{{k}_{adh}^{2}\boldsymbol{ }E}_{F}}{ {E}_{M}} - {v}_{F}^{1/3} \frac{{k}_{adh}^{2}\boldsymbol{ }{E}_{F} - {E}_{M}}{{E}_{M}}}\right).$$

For perfect interfacial adhesion with *k*_adh_ = 1, the Eq. (28) coincides to Ishai-Cohen’s relation, Table 1.

From Eq. (28) also follows that a stiffening effect only occurs if ${k}_{adh}> \sqrt{{E}_{M}/{E}_{F}}$. The filler volume content dependent relative modulus *E*_R_ is calculated with *E*_M_ = 3200 MPa and *E*_F_ = 63,000 MPa, respectively, and varying *k*_adh_ between 0 and 1, Fig. 2b.

From Fig. 2 it is obvious that Eq. (23) produces larger filler volume content dependent Young’s moduli than Eq. (29). Introducing the adhesion factor *k*_adh_ causes a decrease of Young’s moduli. Therefore, only Eq. (23) representing the upper bound is used to derive adhesion factors from measured Young’s moduli.

## Materials and methods

### Materials

Five kinds of polymer matrices filled with various contents of glass beads (GB) as spherical inclusions were used in this study (Table 2):

- polyamide 66 (PA66): RADIPOL A45 (0 wt% GB), AKROMID A3 GK 30 1 natur (30 wt% GB), AKROMID A3 GK 40 1 natur (40 wt% GB)
- polybutyleneterephthalate (PBT): Ultradur B 2550 (0 wt% GB), Ultradur B 4300 K4 (20 wt% GB), Ultradur B 4300 K6 (30 wt% GB)
- butadiene rubber (BR): isomer ratio cis:trans:vinyl = 20:60:20, *M*_N_ = 2 to 3 * 10^5^, cross-linked with dicumyl peroxide,0, 15, 30 and 45 vol% GB unsized and sized
- polyethylene (PE-LD): Nova Chemicals LDPE LA-0219-A, Canada with 0, 20, 40, and 60 wt% GB
- isotactic polypropylene (iPP): Petoplen MH418 with 0, 10, 20 and 30 vol% GB

**Table 2:** Properties of matrices and dispersed phase (GB).

Matrix	GB volume content *v*_F_	GB diameter *r*_F_	Matrix density *ρ*_M_	Matrix modulus *E*_M_	Poisson ratio of matrix *µ*_M_	Surface energy^29–31^ *σ*_surface_
	–	µm	g/cm^3^	MPa	–	mJ/m^2^
PA66	0.16/0.23	25	1.14	3100 to 3200	0.42	38 to 55
PBT	0.12/0.19	25	1.30	2600 to 2800	0.41	44 to 49
BR	0.15/0.30/0.45	58	0.95	7.6	0.48	26 to 27
PE-LD	0.08/0.19/0.35	40 to 60	0.93	98	0.48	33 to 35
iPP	0.10/0.20/0.30	Not given	0.91	1070	0.45	31 to 42

The properties of polar PA66 (Technical data sheet, Radici Group, RADIPOL^®^ A45, 2021) and PBT (Technical data sheet; BASF, Ultradur^®^ B 2550–PBT, 2021) composites were determined experimentally, whereas the properties of nonpolar BR^32^, PE-LD^33^ and iPP^34^ were taken from literature. As in Lohrmann^32^ two compound series—one with unsized GB and one with sized GB—were investigated giving the chance to check how surface treatment of GB affects the stiffness. Glass beads filler has density *ρ*_F_ = 2.5 g/cm^3^, Young’s modulus *E*_F_ = 63 GPa and Poisson ratio *µ*_F_ = 0.22.

### Methods

#### Preparation of tensile test bars

Injection molded test bars of PA66 and PBT compounds (type 1A according to ISO 527-2) were tested after annealing for 4 h at 180 °C to minimize effects of a thermal history.

#### Tensile tests

Universal testing machine (Zwick Z100, Zwick/Roell, Ulm, Germany) was used to perform tensile tests according to DIN EN ISO 527-1 with *n* = 5, equipped with a 10 kN load cell (resolution: 0.12%) and a multiXtens extensometer (resolution: 0.1 µm), an initial sample length of 50 mm, a preload of 0.1 MPa. Differing to ISO 527, the tensile tests of PA66 and PBT compounds were performed with a strain rate of 10%/min. The stress–strain-curves were evaluated using the viscoelastic stress strain function (VSSF)^35^:

30 $$\sigma \left(\varepsilon \right) \underbrace { \cong }_{{\varepsilon  < \varepsilon _{R} }}E{\varepsilon }_{R}\left(1-{e}^{-\frac{\varepsilon }{{\varepsilon }_{R}}}\right)$$

with strain rate dependent Young’s modulus *E* and relaxation strain *ε*_R_. Equation (27) is derived from the time dependent solution of the Maxwell model if one substitutes the time by the strain assuming constant strain rates in the tensile test. The fitting procedure was as follows:

- export of raw data of a force *F* and a length change ∆*L*
- error analysis of *F* and ∆*L*
- consideration of an error propagation of a conversion to stress and the strain
- fitting of measured *σ*-*ε*-curves using VSSF
- determination of *E* and *ε*_R_ as well as corresponding standard deviations
- determination of adhesion factors *k*_adh_ of filled compounds using Eq. (18) with *E*_M_ and *E*_F_ as input parameters from Table 2.

The stress–strain curves of BR compounds were taken from^32^, digitized and evaluated correspondingly to include an example of a rubber matrix composite.

#### Determination of adhesion factors

If a filler matrix adhesion is reduced, a composite modulus decreases. As the modulus *E*_EV_ according to the Eq. (18) represents the upper bound for *k*_adh_ = 1, it is used to determine the adhesion factors of the composites depicted in Table 2. The fitting was done for each composite modulus *E*_C_ using the Excel solver tool. In cases of several measured *E*_C_ this allows for providing standard deviations (STD).

#### Scanning electron microscopy (SEM)

Tensile test bars of PA66 and PBT compounds (type 1A according to ISO 527-2) were fractured under cryogenic conditions in a liquid nitrogen after approximately 5 min of storage. The fracture surfaces were sputtered by gold layer for 90 s at 20 mA, 0.1 mbar in an argon atmosphere. SEM (JEOL JSM-IT100, Japan) equipped with a tungsten cathode was used to investigate the structure of the fracture surfaces at 5.0 kV under vaccuum conditions.

#### Finite element analysis (FEA)

**Figure 3:**
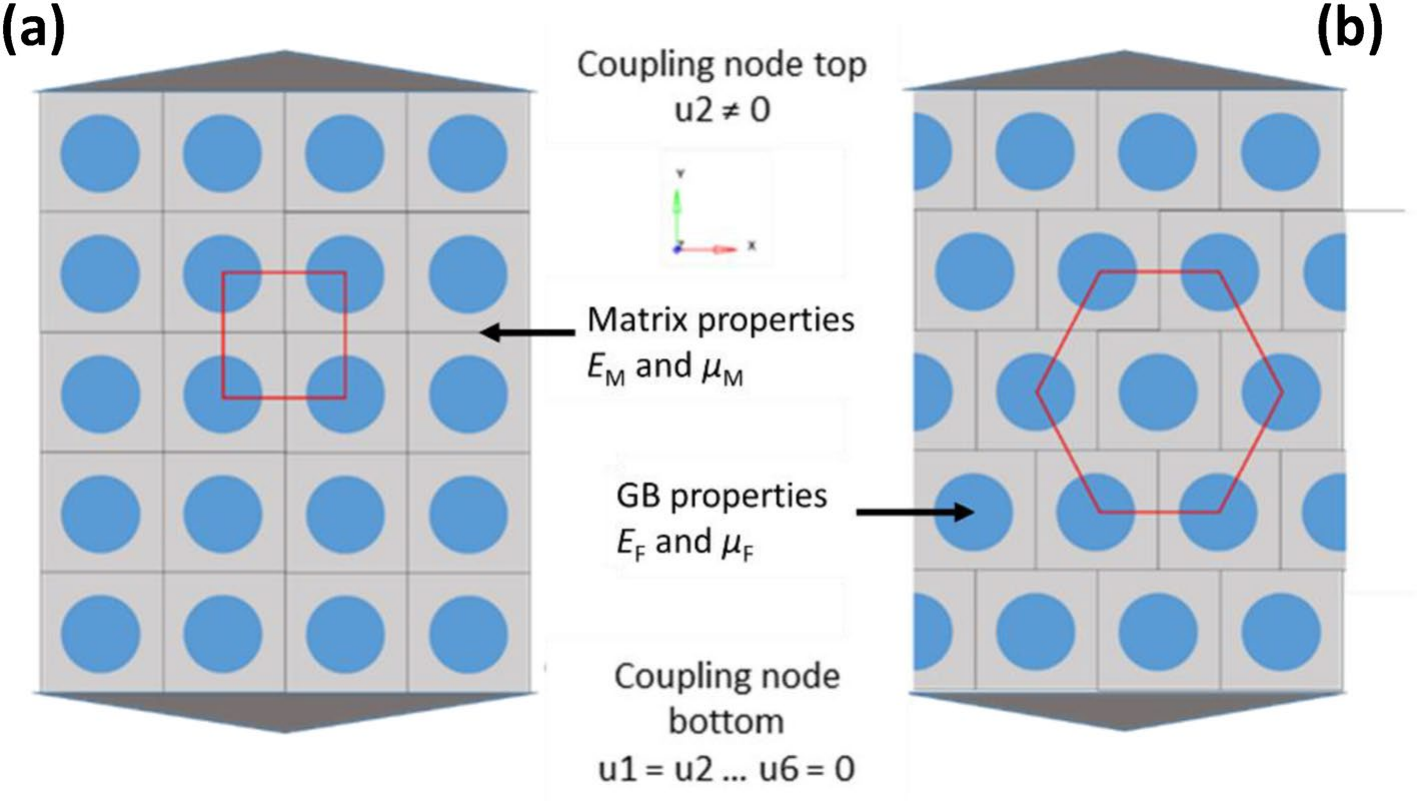
Cubic elementary volume model for FEA simulations: (**a**) cubic and (**b**) hexagonal lattice arrangements. Note that this EV representation holds also for unidirectional fiber systems if the load acts only perpendicular to the fiber axis.

FEA using the software SIMULIA/ABAQUS2020 was performed in 2D for the middle plane of the EV, where maximum stresses occur. A 3D simulation would provide the same stress distribution for the middle plane. A mixed mesh of triangle (S3) and rectangle (S4) shell elements with linear shape functions were used. The total number of elements for each arrangement was approximately 53,000. The nodes at the top and bottom were coupled in *y*- and *z*-directions with a reference node using a kinematic coupling constraint. For the reference node at the bottom all translational and rotational degrees of freedom were fixed to *u*_1_ = *u*_2_ = ….. = *u*_6_ = 0. For the reference node at the top, all degrees of freedom except for the translation in *y*-direction were fixed as well. The displacement in the positive *y*-direction (*u*_2_) was defined at the top node to apply a uniaxial tensile load. Static linear analysis was carried out by implementing materials properties as a filler modulus *E*_F_ and a matrix modulus *E*_M_, a filler Poisson ratio *µ*_F_ and a matrix Poisson ratio *µ*_M_, and a filler volume content *v*_F_. Filler content dependent EV arrangements in a cubic and a hexagonal lattice were investigated, Fig. 3. Maximum achievable filler contents are ${v}_{F,\mathrm{max}}=\pi /6\approx 0.52$ for cubic and ${v}_{F,\mathrm{max}}=\pi /\sqrt{27}\approx 0.60$ for hexagonal arrangements. For given technically relevant filler volume contents below 0.4 both arrangements are identical with respect to a space filling but not to a force flow under uniaxial loads.

## Results and discussion

The tensile properties of PA 66 and PBT composites were determined according to ISO 527 and evaluated using the VSSF, Table 3. Both PA66 and PBT compounds showed a stiffening effect with increasing GB contents, whereas reinforcement is only found for the PA66 compounds indicating a higher filler matrix adhesion. Moduli determined by ISO 527 are smaller than those determined by the VSSF because the strain rate of 10%/min is not reached yet at strains *ε*_1_ = 0.05% and *ε*_2_ = 0.25%.

**Table 3:** Tensile properties of composites.

Matrix	Filler content *v*_F_	Modulus *E*_ISO 527_	Tensile strength *σ*_max_	Tensile elongation *ε*_max_	Modulus *E*_VSSF_	Relaxation strain *ε*_*R*_
	–	MPa		%	MPa	%
PA66	0	3143 ± 14	81 ± 0.3	3.9 ± 0.5	3197 ± 28	9.4 ± 0.2
PA66	0.16	4811 ± 22	91 ± 1.1	3.1 ± 0.8	4955 ± 44	7.8 ± 0.1
PA66	0.23	5299 ± 45	86 ± 0.2	3.7 ± 0.5	5452 ± 48	7.2 ± 0.1
PBT	0	2704 ± 18	59 ± 0.1	10.8 ± 1.4	2754 ± 25	9.4 ± 0.1
PBT	0.12	3581 ± 32	57 ± 0.1	3.2 ± 0.2	3691 ± 33	5.9 ± 0.1
PBT	0.19	4254 ± 30	55 ± 0.1	2.0 ± 0.6	4408 ± 39	5.4 ± 0.2
BR_unsized_	0	–	–	–	7.0	6.2
	0.15	–	–	–	11.0	6.3
	0.30	–	–	–	16.8	8.0
	0.45	–	–	–	48.0	0.7
BR_sized_	0	–	–	–	6.9	5.0
	0.15	–	–	–	9.9	4.6
	0.30	–	–	–	19.2	5.5
	0.45	–	–	–	52.2	1.1
PE-LD	0	–	8.4 ± 0.2	–	98 ± 5	–
	0.15	–	7.8 ± 0.5	–	124 ± 6	–
	0.30	–	6.2 ± 0.3	–	175 ± 27	–
	0.45	–	3.3 ± 0.1-	–	219 ± 76	–
iPP	0	–	31.1 ± 0.5	14.7 ± 0.4	1070 ± 20	–
	0.10	–	26.0 ± 1.7	10.6 ± 0.8	1230 ± 10	–
	0.20	–	18.8 ± 4.9	6.1 ± 1.6	1280 ± 20	–
	0.30	–	15.9 ± 1.3	45 ± 1.2	1310 ± 20	–

For BR compounds the standard deviation could not be determined because there was only one stress strain curve for each compound available in Lohrmann^32^. For PE-LD and iPP the data were taken directly from Chimeni et al.^33^ and Balkan and Demirer^34^.

The adhesion factors *k*_adh_ were determined by a fitting procedure that brings calculated moduli *E*_EV_ according to the Eq. (18) to coincidence to measured moduli *E*_VSSF_. The comparison of *E*_EV_ to calculated moduli *E*_C_ according to Ishai-Cohen, Paul and Halpin–Tsai with *ξ* = 2 for the transversal case shows that fitting the composites moduli according to Eq. (18) provides reasonable values of the adhesion factors, Table 4. It confirms that Paul and Ishai-Cohen models represent the upper and lower bounds, respectively. The calculated moduli according to Halpin–Tsai provide reasonable *E*_C_ values, but the efficiency factor *ξ* is hardly related to filler matrix adhesion as the calculated *E*_C_ can be larger or smaller compared to the measured ones.

**Table 4:** Comparison of calculated GB content dependent Young’s moduli with evaluated adhesion factors *k*_adh_ and its minimal stiffening limit ${k}_{adh, min}^{stiffening}=\sqrt{{E}_{M}/{E}_{F}}$.

Matrix	Filler content *v*_F_	Calculated Young’s modulus *E*_*C*_				Adhesion factor *k*_adh_	Stiffening limit ${k}_{adh, min}^{stiffening}$
		Ishai-Cohen	Paul	Halpin–Tsai	EV *(E*_EV_ ≡ *E*_VSSF_*)*		
	–	MPa					–
PA66	0.16	4229 ± 35	5963 ± 44	4770 ± 32	4955 ± 44	0.604 ± 0.017	0.225
	0.23	4899 ± 40	6940 ± 51	5408 ± 36	5452 ± 48	0.570 ± 0.013	
PBT	0.12	3370 ± 30	4744 ± 37	3754 ± 26	3691 ± 33	0.478 ± 0.015	0.209
	0.19	3895 ± 34	5608 ± 43	4449 ± 30	4408 ± 39	0.530 ± 0.013	
BR^unsized^	0.15	9.2	14.9	10.7	11.0	0.031	0.011
	0.30	13.3	21.1	16.0	16.8	0.042	
	0.45	20.4	29.9	24.1	29.9^a^	n.f	
BR^sized^	0.15	9.2	14.8	10.6	9.9	0.025	0.011
	0.30	13.2	21.0	15.8	19.2	0.073	
	0.45	20.3	29.6	23.9	29.7^a^	n.f	
iPP	0.10	1263	1876	1407	1230	0.220	0.130
	0.20	1565	2414	1824	1280	0.190	
	0.30	1993	3010	2351	1310	0.180	
PE-LD	0.08	112	173	125	124	0.100	0.039
	0.19	143	232	167	175	0.130	
	0.35	215	331	257	219	0.110	

^a^To calculate *E*_EV_ the adhesion factor was set to *k*_adh_ = 1. n.f. means “no fit available”.

For the polar polymer matrices PA66 and PBT, *k*_adh_ is found to be around 0.6 and 0.5, respectively, whereas it is smaller than 0.25 for the nonpolar polymer matrices iPP, PE-LD and BR, which is in accordance with the surface energies shown in Table 1. In spite of rather low *k*_adh_ all analyzed composites exhibit stiffening due to GB introduction as they always exceed the stiffening limit.

**Figure 4:**
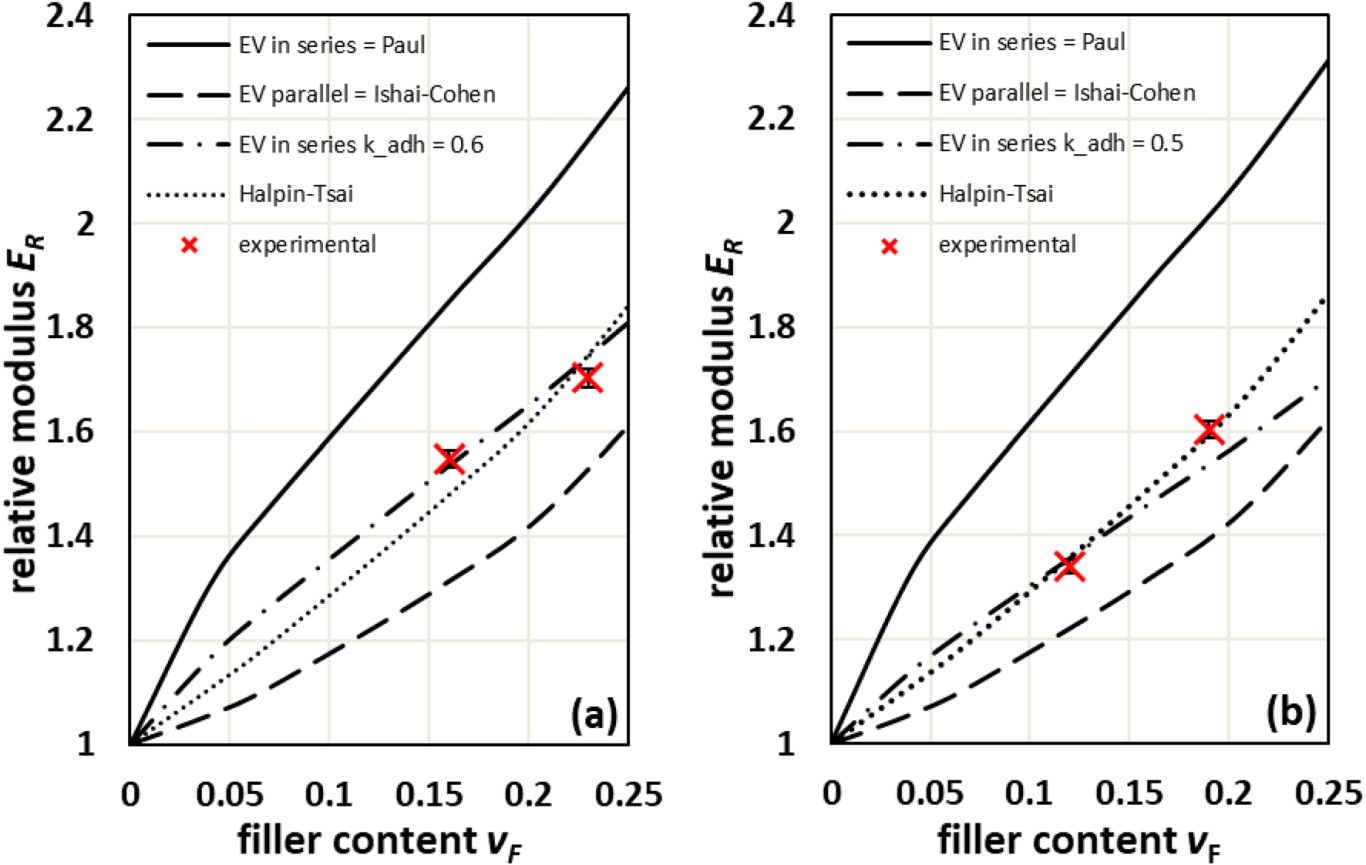
Comparison of relative Young’s moduli calculated according to Ishai-Cohen, Paul, Halpin–Tsai with *ξ* = 2, and EV for *k*_adh_ = 0.5 and 0.6 (lines) with measured ones (symbols) for (**a**) PA66 and (**b**) PBT.

The comparison of measured Young’s moduli of GB filled PA66 and PBT composites to calculated ones, Fig. 4, confirms that the models of Ishai-Cohen and Paul represent the lower and the upper bounds. The measured Young’s moduli lie between these bounds as well as the calculated moduli according to Halpin–Tsai with *ξ* = 2 and EV with *k*_adh_ = 0.6 (PA66) and 0.5 (PBT). Both reproduce well the measured Young’s moduli, but only the EV provides the quantitative information concerning the filler-matrix adhesion.

**Figure 5:**
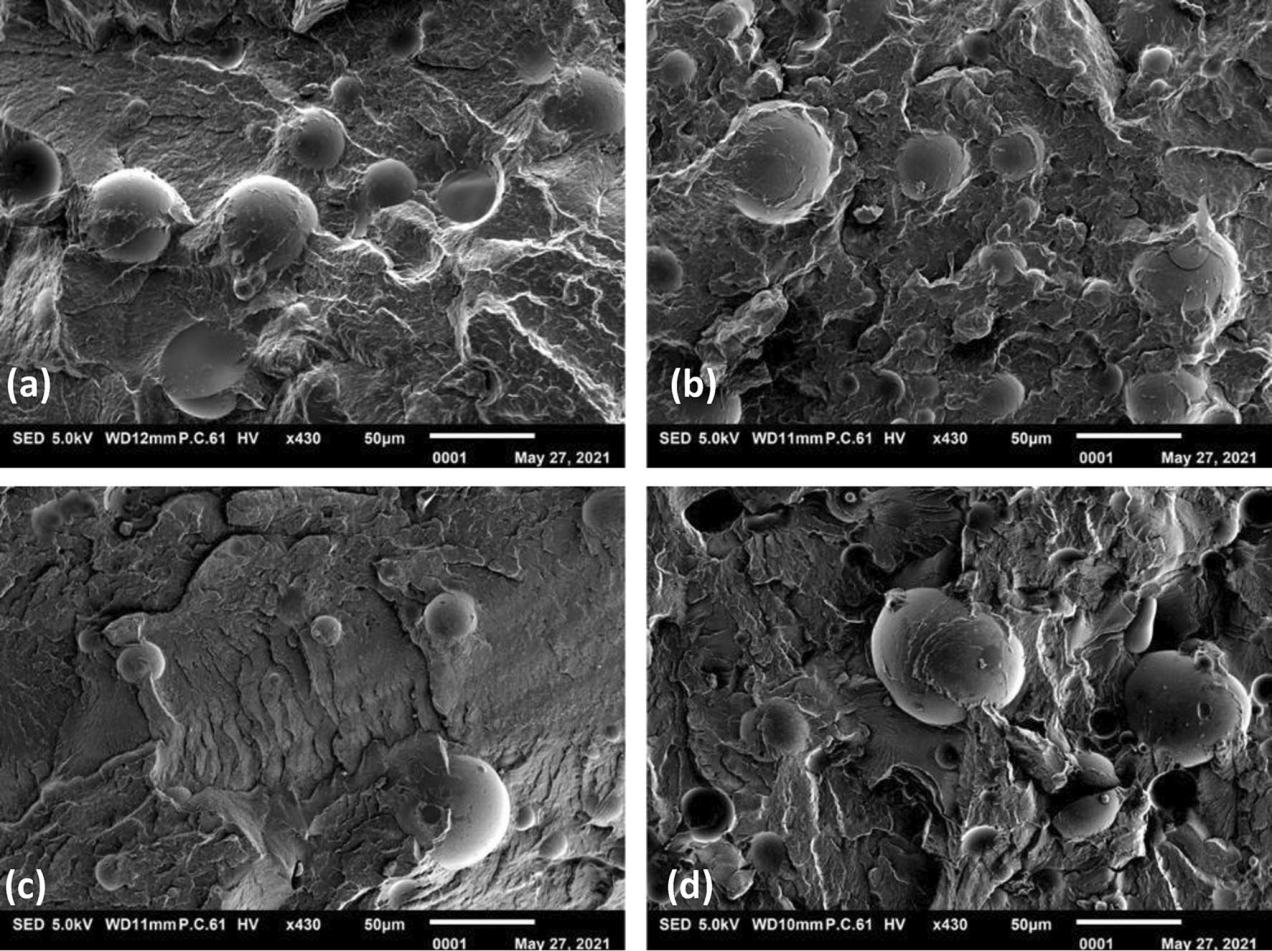
Fracture surface of composites: (**a**) PA66/GB16, (**b**) PA66/GB23, (**c**) PBT/GB12 and (**d**) PBT/GB19.

SEM images of PA66 and PBT composites show a fracture surface with embedded GB, which are partly covered by the matrix indicating rather poor filler-matrix adhesion, Fig. 5.

Better adhesion seen for the PA66 composite correlates with its higher *k*_adh_. Small fibrils partly visible on the fractured surfaces of both the PA66 and PBT composites correspond to ductile failure of the matrices. It seems that the fracture behavior becomes more ductile, the higher the GB content is.

Similar increases of filler content dependent Young’s moduli of elastomer (BR) composites were found for all models, Table 4, showing a stiffening effect although the adhesion factors become small. The introduction of a coupling agent to one BR composite has hardly an effect. The Halpin–Tsai model produces similar Young’s moduli as the EV with *k*_adh_ between 0.02 and 0.05 for *v*_F_ = 0.15 and 0.30. For *v*_F_ = 0.45 all models generate too small Young’s moduli. This can be attributed to the fact that at high filler contents, particle interactions become stronger going along with the formation of a particle network with significantly higher modulus^36^. On first sight this issue could be eliminated by allowing adhesion factors exceeding 1. However, it turned out that the models of Ishai-Cohen and Paul limit the filler volume content dependent stiffening factors to:

31 $$\mathrm{Ishai}{-}\mathrm{Cohen } \quad s\left({v}_{F}\right)= \frac{{E}_{ EV}}{{E}_{ M}} = \frac{{v}_{F} }{ 1 - {v}_{F}^\frac{1}{3} } \underbrace { \longrightarrow }_{{v_{F}  = 0.45}} \;\; 2.9$$

32 $$\mathrm{Paul} \quad s\left({v}_{F}\right)= \frac{{E}_{ EV}}{{E}_{ M}} = \frac{1 }{ 1 - {v}_{F}^\frac{1}{3} } \underbrace { \longrightarrow }_{{v_{F}  = 0.45}} \;\; 4.3$$

**Figure 6:**
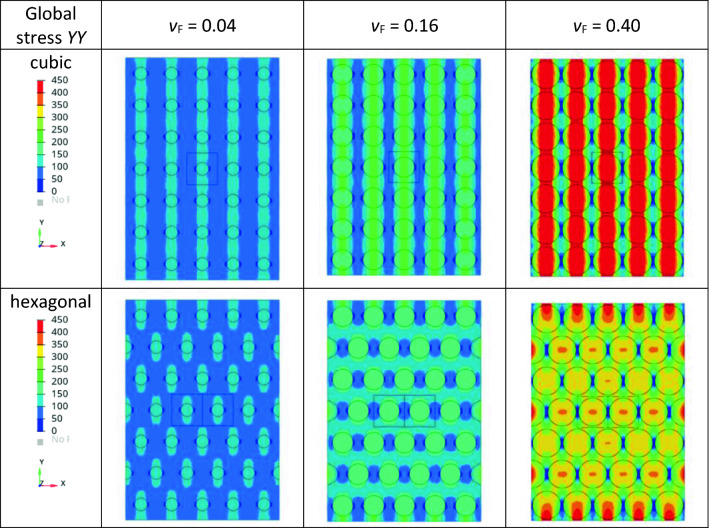
Filler content dependent stress distribution in tensile direction (*YY*) of cubic and hexagonal lattice arrangements of EV under tensile load in *y*-direction. Note that the displacements in *y*-direction are represented in a magnified manner.

From Fig. 3 it is obvious that the cubic arrangement of EV leads to less shear stresses compared to the hexagonal arrangement although the only difference is that each 2nd layer of EV is shifted by one half of the edge length. This is confirmed by purely elastic FEA simulations showing that the stress distributions differ significantly for the cubic and hexagonal EV arrangement, Fig. 6. The stresses have to be interpreted with respect to tensile strengths of matrix and GB, whereas *σ*_y,GB_ >  > *σ*_y,M_. For all cubic arrangements the maximum stresses occur between the GB in the matrix forming the parallel stress lines in the composite part along the load direction, whereas in the interjacent matrix parts the stresses remain smaller. With the increasing filler content, the stresses in tensile direction increase leading to a failure of the interface, and cracks propagation perpendicular to the load. Nevertheless, the stresses in both parts are always oriented in the parallel and uniaxial manner in tensile direction.

The hexagonal arrangement of EV shows stress pattern with in-load direction oriented oval “stress islands” around the GB particles. The minimum stresses occur perpendicular to the load direction in the matrix between the GB. The matrix stresses in the load direction between the GB seem to be more homogeneous compared to the cubic arrangement providing more deformability. With increasing the GB content, the stress islands unify and form shear stress band under an angle of 45° seen in Fig. 6—hexagonal/*v*_F_ = 0.40. Both reduces moduli of the hexagonal arrangement in the load direction, Table 5, but the difference remains less than 10% for volume filler contents not exceeding 20%.

**Table 5:** FEA calculated filler volume content dependent Young’s moduli of cubic and hexagonal lattice arrangements of EV with *E*_M_ = 3200 MPa, *E*_F_ = 63,000 MPa, *µ*_M_ = 0.42 and *µ*_F_ = 0.22.

Filler content, *v*_F_	*E*_C_ (cubic lattice)	*E*_C_ (hexagonal lattice)	*E*_C_ ratio cubic:hexagonal
–	MPa	MPa	
0.04	3950	3914	1.01
0.07	4389	4308	1.02
0.16	5988	5656	1.06
0.22	7461	6827	1.09
0.40	14,534	11,632	1.25

From Table 5 one can conclude that the properties of the EV are representative of composites properties for filler volume contents not exceeding 15 to 20%. In this filler content range, the EV arrangement effects remain in the order of the experimental error. For higher filler contents, the arrangement of EV starts to affect the composites moduli due to increasing shear stresses. The two considered EV arrangements are boundary cases. The real EV arrangements lie rather in between, and thus the measured moduli should be smaller than those of the cubic arrangement, but larger than those of the hexagonal arrangement. It means that the adhesion factor *k*_adh_ is under-estimated if the Eq. (18) is used for fitting.

Increasing of filler content ease crystallization for semi-crystalline polymers due to more nucleation sites. Thus, matrix moduli are increased due to higher crystallinities^9^. However, higher matrix moduli have the consequence that the adhesion factors *k*_adh_ are determined slightly smaller. This shows that adhesion factors are also affected by the choice of the matrix polymer.

## Conclusion

The *cube in cube* models of Paul (upper bound) and Ishai-Cohen (lower bound) determine the moduli of particle filled composites assuming perfect filler-matrix adhesion. To take into account reduced adhesion between filler particles and matrix polymer their models were recalculated using an EV approach, and extended by an adhesion factor *k*_adh_ that scales the edge length of the cubic inclusion. It leads to the reduction of the filler content dependent composites moduli *E*_C_(*v*_F_). The modified Paul model in EV version as the upper bound of *E*_C_(*v*_F_) was used to fit experimental moduli of glass bead filled polymers and provided reasonable adhesion factors: 0.6 for polyamide (PA66), 0.5 for polybutylene terephthalate (PBT), 0.2 for polypropylene (iPP), 0.13 for low density polyethylene (PE-LD) and 0.05 for butadiene rubber (BR), which are in line with the corresponding surface energies. The modified model allows for design engineers to calculate realistic modulus of any particulate composite using only matrix and filler moduli, filler content and adhesion factor without performing tests. Further analysis of the modified model elucidated that stiffening only occurs if *k*_adh_ exceeds $\sqrt{{E}_{M}/{E}_{F}}$ and relates stiffening to the ratio of matrix modulus *E*_M_ and filler modulus *E*_F_. This means almost all technical relevant filler contents lead to stiffening of the polymer. Furthermore, the EV approach shows directly that the moduli of particulate composites depend on particle shape—quantified by the efficiency factor *k*—and the dimensions within the EV—expressed by the normalized filler distance *d* which depends on a filler volume content.

Additionally, finite element analyses of cubic and hexagonal EV arrangements show that the spatial EV arrangement leads to differences of the filler content dependent composites moduli especially for high volume contents. Thus, the assumption “The properties of the EV are representative for the whole composites” only holds for filler volume contents up to 15 or 20%. The increasing difference between composites moduli calculated for cubic and hexagonal EV arrangement can be attributed to two limits of the *cube in cube* model: first, the occurrence of increasing shear stresses if EV arrangements deviate from cubic arrangements, and second, increasing interaction among filler particles with the formation of particle networks within the matrix disturbing the EV assumption of homogeneously dispersed particles. The modulus increase of rubber composite by factor 2 or 3 if the filler content changes from 30 to 45%, respectively, cannot be explained within the *cube in cube* model anymore. From a practical point of view, where commercially available particle filled polymers have filler volume contents less than 20%, it is positive that the EV arrangement affects calculated composites moduli within the experimental error of 3 to 5%.

## Supplementary Information

Supplementary Information.

## Data Availability

The datasets generated during and/or analysed during the current study are available in the Zenodo open repository maintained by https://zenodo.org/record/6460262.

## References

[1] Thomas S (2012). Polymer Composites.

[2] Fu SY, Feng XQ, Lauke B, Mai YW (2008). Effects of particle size, particle/matrix interface adhesion and particle loading on mechanical properties of particulate-polymer composites. Compos. Part B-Eng..

[3] He D, Jiang B (1993). The elastic modulus of filled polymer composites. J. Appl. Polym. Sci..

[4] Demir H, Atikler U, Balköse D, Tihminlioglu F (2006). The effect of fiber surface treatments on the tensile and water sorption properties of polypropylene-luffa fiber composites. Compos. Part A-Appl. Sci. Manuf..

[5] Jacob M, Francis B, Varughese KT, Thomas S (2006). The effect of silane coupling agents on the viscoelastic properties of rubber biocomposites. Macromol. Mater. Eng..

[6] Ku H, Wang H, Pattarachaiyakoop N, Trada M (2011). A review on the tensile properties of natural fiber reinforced polymer composites. Compos. Part B-Eng..

[7] Dekkers MEJ, Heikens D (1983). The effect of interfacial adhesion on the tensile behavior of polystyrene–glass-bead composites. J. Appl. Polym. Sci..

[8] Dibendetto AT, Wambach AD (1972). The fracture toughness of epoxy-glass-bead composites. Int. J. Polym. Mater..

[9] Wang K, Wu J, Ye L, Zeng H (2003). Mechanical properties and toughening mechanisms of polypropylene/barium sulfate composites. Compos. Part A-Appl. Sci. Manuf..

[10] Hill R (1963). Elastic properties of reinforced solids: Some theoretical principles. J. Mech. Phys. Solids.

[11] Hashin Z, Shtrikman S (1963). A variational approach to the theory of the elastic behavior of multiphase materials. J. Mech. Phys. Solids.

[12] Christensen RM, Lo KH (1979). Solutions for effective shear properties in three phase sphere and cylinder models. J. Mech. Phys. Solids.

[13] Zare Y (2015). Assumption of interphase properties in classical Christensen-Lo model for Young’s modulus of polymer nanocomposites reinforced with spherical nanoparticles. RSC Adv..

[14] Zare Y (2016). Development of Halpin-Tsai model for polymer nanocomposites assuming interphase properties and nanofiller size. Polym. Test..

[15] Voigt W (1889). Ueber die Beziehung zwischen den beiden Elasticitätsconstanten isotroper Körper. Ann. Phys..

[16] Reuss A (1929). Berechnung der Fließgrenze von Mischkristallen auf Grund der Plastizitätsbedingung für Einkristalle. Zamm-Z Angew Math Me.

[17] Guth E (1945). Theory of filler reinforcement. J. Appl. Phys..

[18] Einstein A (1906). Eine neue Bestimmung der Moleküldimensionen. Ann. Phys..

[19] Takayanagi M, Uemura S, Minami S (1964). Application of equivalent model method to dynamic rheo-optical properties of crystalline polymer. J. Polym. Sci. Pol. Sym..

[20] Paul B (1960). Prediction of constants of multiphase materials. Trans. Am. Inst. Min. Metall. Pet. Eng..

[21] Ishai O, Cohen IJ (1967). Elastic properties of filled and porous epoxy composites. Int. J. Mech. Sci..

[22] Hirsch TJ (1962). Modulus of elasticity of concrete affected by elastic moduli of cement paste matrix and aggregate. Amer. Conc..

[23] Counto UJ (1964). The effect of the elastic modulus of the aggregate on the elastic modulus, creep and creep recovery of concrete. Mag. Concrete Res..

[24] Halpin, J. C. & Tsai, S. W. Effects of environmental factors on composite materials. *Technical Report. AFML-TR,* 67–423 (1969).

[25] Nielsen LE (1970). Generalized equation for the elastic moduli of composite materials. J. Appl. Phys..

[26] Lewis TB, Nielsen LE (1970). Dynamic mechanical properties of particulate filled composites. J. Appl. Polym. Sci..

[27] Nielsen LE (1979). Dynamic mechanical properties of polymers filled with agglomerated particles. J. Appl. Polym. Sci..

[28] Kerner EH (1956). The elastic and thermo-elastic properties of composite media. Proc. Phys. Soc. B.

[29] Kopczynska, A. & Ehrenstein, G. W. Oberflächenspannung von Kunststoffen. Messmethoden am LKT. Sonderdrucke am Lehrstuhl für Kunststofftechnik, Friedrich-Alexander-Universität Erlangen-Nürnberg, https://www.lkt.tf.fau.de/files/2017/06/Oberflaechenspannung.pdf (2017).

[30] Erhard, G. Konstruieren mit Kunststoffen. 4. Auflage, 152–153 (Carl Hanser Verlag, 2008).

[31] Ansafirar A, Critchlow GW, Guo R, Ellis RJ, Haile-Meskel Y, Doyle B (2009). Assessing effect of the migration of a paraffin wax on the surface free energy of natural rubber. Rubber Chem. Technol..

[32] Lohrmann, M. Zweidimensionale Ermittlung der Änderung des mechanischen Verhaltens von gefüllten Elastomeren bei Variation der Füllstoffart. *Fraunhofer-Institut für Chemische Technologie ICT* (1996).

[33] Chimeni DY, Vallée E, Sorellis L, Rodrigue D (2018). Effect of glass bead size and content on the thermomechanical properties of polyethylene composites. Polym. Eng. Sci..

[34] Balkan O, Demirer H (2010). Mechanical properties of glass bead- and wollastonite-filled isotactic-polypropylene composites modified with thermoplastic elastomers. Polym. Compos..

[35] Möginger B, Fritz U (1994). Viskoelastische Spannungs-Dehnungs-Beziehung thermoplastischer Polymere: Herleitung und experimentelle Überprüfung. Kautschuk und Gummi Kunststoffe.

[36] Kashfipour MA, Mehra N, Zhu J (2018). A review on the role of interface in mechanical, thermal, and electrical properties of polymer composites. Adv. Compos. Hybrid Mater..

