# Supplementary material for: Introduction of an adhesion factor to cube in cube models and its effect on calculated moduli of particulate composites
Source: Sci Rep. 2022 Sep 28;12:16225. doi: 10.1038/s41598-022-20629-2 (PMC9519868; doi:10.1038/s41598-022-20629-2)
Supplement: Supplementary file 1 — Supplementary Information. [file 41598_2022_20629_MOESM1_ESM.pdf]

Appendix A – Relation between macroscopic and microscopic strains

The "macroscopic" strain of the EV ε_{EV} has to depend on the microscopic strains of matrix part ε_M and composites part ε_C. It also has to be affected by the dimensions of the EV in relation to the inclusion. Thus, one starts with the definition of the strain of the EV with spherical inclusion and its transformation in the *cube in cube* representation:

$$\varepsilon_{EV} = \underbrace{\frac{\Delta(D+a)}{D+a}}_{sphere\ in\ EV} = \underbrace{\frac{\Delta(kD+(D-kD)+a)}{kD+(D-kD)+a}}_{cube\ in\ EV} = \frac{\Delta(kD)+\Delta(D-kD+a)}{kD+(D-kD)+a} \tag{A1}$$

After separation of the stress induced length change into the length changes of the inclusion/cube and matrix, one gets:

$$\varepsilon_{EV} = \frac{\Delta(kD)}{kD+(D-kD)+a} + \frac{\Delta(D-kD+a)}{kD+(D-kD)+a} = \frac{\frac{\Delta(kD)}{kD}}{\frac{kD+(D-kD)+a}{kD}} + \frac{\frac{\Delta(D-kD+a)}{(D-kD)+a}}{\frac{kD+(D-kD)+a}{(D-kD)+a}} \tag{A2}$$

Multiplying the first fraction with *kD* and the second fraction with *D-kD+a* allows for the introduction of the microscopic strains. After cancelation of *D* one gets:

$$\varepsilon_{EV} = \frac{\frac{\Delta k}{k}}{\frac{k+(1-k)+d}{k}} + \frac{\frac{\Delta(1-k+d)}{(1-k)+d}}{\frac{k+(1-k)+d}{(1-k)+d}} = \frac{\varepsilon_C}{\frac{k+(1-k)+d}{k}} + \frac{\varepsilon_M}{\frac{k+(1-k)+d}{(1-k)+d}} \tag{A3}$$

Rearranging leads to equation (20):

$$\varepsilon_{EV}(\varepsilon_M, \varepsilon_C) = \frac{1-k+d}{1-d}\varepsilon_M + \frac{k}{1-d}\,\varepsilon_C = \left(1-\frac{k}{1-d}\right)\varepsilon_M + \frac{k}{1-d}\,\varepsilon_C \tag{A4}$$

showing that microscopic strains are weighted by factors which depend on particle shape and EV dimensions. It must be stressed that this distinguishing is lost if equation (22) is used to introduce the filler volume content:

$$\varepsilon_{EV}(\varepsilon_M, \varepsilon_C) = \left(1-v_F^{1/3}\right)\varepsilon_M + v_F^{1/3}\,\varepsilon_C \tag{A5}$$
